# Dual-Stage Annealing for Enhanced Thulium Oxynitride Passivation on a 4H-SiC MOS Capacitor

**DOI:** 10.1021/acsomega.6c01056

**Published:** 2026-03-27

**Authors:** Junchen Deng, Jianhong Chen, Hock Jin Quah

**Affiliations:** † School of Electronic Engineering, 518871 Lanzhou City University, Lanzhou 730070, China; ‡ Institute of Nano Optoelectronics Research and Technology (INOR), 26689 Universiti Sains Malaysia, 11800, Penang, Malaysia

## Abstract

Enhancement of the metal-oxide-semiconductor (MOS) characteristics for a thulium oxynitride (Tm_*x*_O_*y*_N_*z*_) passivation layer (PL) on 4H-silicon carbide (SiC) was achieved through the development of a dual-stage process. This methodology comprised rapid thermal annealing in a nitrogen (N_2_) ambient, followed by normal annealing (NA) in a forming gas-oxygen-forming gas (FOF) mixture. Analysis by grazing incidence X-ray diffraction (GIXRD) and X-ray photoelectron spectroscopy (XPS) confirmed the successful formation of the Tm_*x*_O_*y*_N_*z*_ PL. The additional RTA step enhanced nitrogen incorporation at the Tm_*x*_O_*y*_N_*z*_/4H-SiC interface, effectively reducing oxygen vacancies (*V*_o_). Data from X-ray reflectivity (XRR) and cross-sectional FESEM corroborated these findings, indicating that the accumulation of nitrogen ions facilitated the development of a thinner interfacial SiO_2_ layer with a thickness of 2.422 nm. Consequently, the electrical properties of the dual-stage annealed Tm_*x*_O_*y*_N_*z*_ PL were enhanced with a higher dielectric constant (*k* = 13.1), lower slow trap density (STD = 4.24 × 10^11^ cm^–2^), lower interface trap density (*D*_it_), and lower leakage current density (*J*).

## Graphical abstract

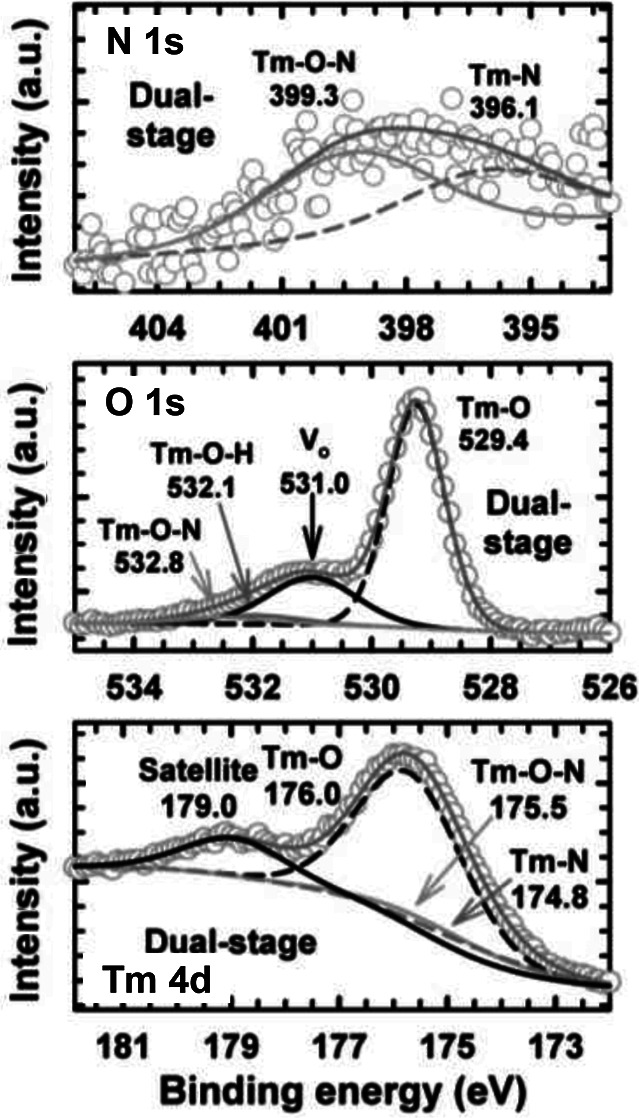

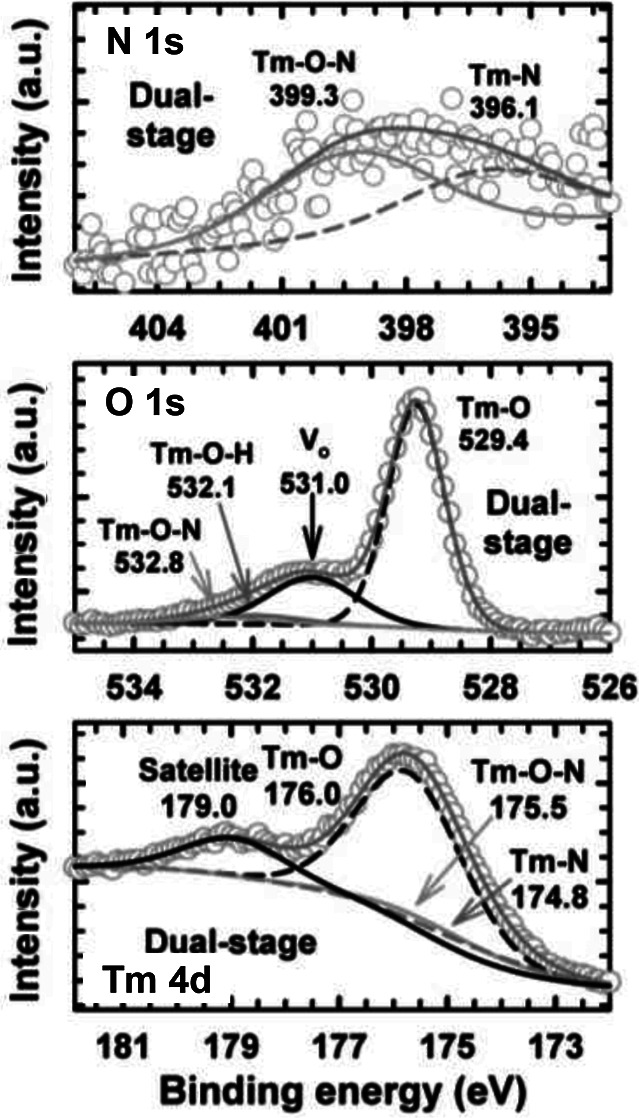

## 1 Introduction

The growing share of renewable energy in the global supply has spurred demand for advanced power electronics capable of meeting the high power and temperature requirements of modern energy storage and transmission.^1^ For these applications, silicon carbide (SiC), especially the 4H-SiC polytype, is ideal because of its high breakdown field (*E*_B_ = 3.0 MV/cm), wide bandgap (*E*_g_ = 3.26 eV), superior thermal conductivity (4.9 W/cm·K), and low intrinsic carrier concentration (∼10^–9^ cm^3^), enabling operation in harsh environments.^2–4^ The progression of metal-oxide-semiconductor (MOS) devices utilizing 4H-SiC substrates was substantially hindered by the material properties of conventional silicon dioxide (SiO_2_) PL, particularly their low dielectric constant (*k* = 3.9).^5^ The fundamental principles of Gauss’s law established that the breakdown characteristics of the entire device were constrained by the breakdown field of the SiO_2_ layer.^6^ Addressing these limitations was essential to harness the superior breakdown field of 4H-SiC. Consequently, significant investigative efforts were directed toward employing high-*k* dielectrics as PL. Candidate materials that were explored for this role included HfO_2_,^7^ Al_2_O_3_,^8^ AlN,^9^ ZrO_2_,^10^ La_2_O_3_,^11^ Y_2_O_3_,^12^ CeO_2_,^13^ and Tm_2_O_3_.^14^ It was highlighted in recent work that the performance of 4H-SiC MOS-based devices in terms of specific on-resistance, gate-drain capacitance, and breakdown voltage was influenced by the quality of PL and its interface with the 4H-SiC substrate.^15^

Among various high-*k* materials, Tm_2_O_3_ stands out with a theoretical *k* value of 12.77 ^16^ and a large *E*_g_ of 5.15 eV,^17^ making it a promising candidate as PL for 4H-SiC-based MOS devices. Recent studies have reported the feasibility of using Tm_2_O_3_ as a PL on the 4H-SiC substrate. When annealed in an FG-O_2_-FG ambient at 700 °C, the Tm_2_O_3_ PL exhibited a high *k* value of 9.9, a high *E*_B_ of 3.4 MV/cm, and a moderate interface states density (*D*_it_) of 1.51 × 10^12^ eV^–1^cm^–2^ at an energy level of 0.4 eV.^14^ Nevertheless, the obtained *k* and *E*_B_ values were lower than those reported for Tm_2_O_3_ PL on Si substrates, which exhibited a *k* of 18.0 ^18^ and an *E*_B_ of 4.0 MV/cm.^19^ This suggested that the passivating properties of Tm_2_O_3_ PL on the 4H-SiC substrates could be further unlocked through further optimization of the annealing processes. Studies of other high-*k* materials on 4H-SiC substrate have shown that a two-step annealing process, using different temperatures and ambients, can improve MOS characteristics.^20,21^ It was reported that atomic layer deposited Al_2_O_3_ PL on 4H-SiC substrate annealed at 1100 °C in an oxygen ambient followed by the subsequent annealing process at 400 °C in FG ambient has led to improvements in *E*_B_, narrowing of capacitance–voltage (*C*–*V*) hysteresis, and a decrease in *D*_it_.^20^ Besides, improvement in *D*_it_ was observed for the Al_2_O_3_/4H-SiC structure annealed at 600 °C in nitrogen ambient for 30 s, with an additional annealing step at 400 °C in FG ambient for 30 min, compared to a sample subjected to a single-stage annealing process.^21^ Another novel technique of improving the electrical performance, including reduced *D*_*it*_, smaller *C*–*V* hysteresis, lower leakage current density (*J*), and a higher *E*_*B*_, as well as suppressing the growth of a detrimental SiO_*x*_ interfacial layer (IL), was through the introduction of a lanthanum silicate (LaSiO_*x*_) interlayer between the Al_2_O_3_ PL and the 4H-SiC substrate.^22^ In this work, a similar beneficial modification of the interface using a dual-stage annealing process was proposed for the deposited Tm_*x*_O_*y*_N_*z*_ PL on a 4H-SiC substrate without introducing a distinct interfacial material. Therefore, this letter presents a systematic study of the Tm_*x*_O_*y*_N_*z*_ PL sputtered on a 4H-SiC substrate after a dual-stage annealing process involving RTA coupled with NA.

## 2 Experimental Procedure

The n-type 4H-SiC (0001) wafer was diced into smaller samples (1 cm × 1 cm), cleaned using the method recommended by Radio Corporation of America (RCA), and then dipped in diluted hydrofluoric acid. The deposition of Tm_2_O_3_ PL was carried out using an RF magnetron sputtering system (HVV AUTO A500) at 100 W RF power for 40 min, with 15 sccm of argon gas flow rate and 7.6 × 10^–3^ mbar of working pressure. The as-deposited sample underwent RTA at 800 °C in nitrogen (N_2_) ambient for 3 min, followed by NA at 800 °C in FG-O_2_-FG ambient for 30 min, while another sample was subjected to only NA. During the RTA process, the N_2_ flow rate was 30 sccm, while the flow rates of FG, oxygen (O_2_), and FG during the heating, cooling, and dwelling stages, respectively, during the NA process were all 100 sccm. The top aluminum (Al) contacts with a diameter of 0.2 mm were deposited on the investigated Tm_*x*_O_*y*_N_*z*_ PL using a thermal evaporator (AUTO 306) and a shadow mask. The same method was then used to deposit a blanket Al contact on the backside of the 4H-SiC substrate. XPS (Thermo Fisher Scientific Nexsa) using a monochromatic Al K_α_ X-ray source (hv = 1486.6 eV) at 150 W and a 30° emission angle was used to analyze the chemical states of the studied Tm_*x*_O_*y*_N_*z*_ PL. Charging effects were corrected by referencing the C 1s peak (284.6 eV) of adventitious carbon. GIXRD (Bruker D8 Discover) was used to characterize the structures of the materials under investigation. The thickness of the investigated Tm_*x*_O_*y*_N_*z*_ PL was characterized using cross-sectional field-emission scanning electron microscopy (FESEM; FEI Nova Nano SEM 450), while X-ray reflectivity (XRR; Bruker D8 Discover) was used to determine the thickness of the investigated Tm_*x*_O_*y*_N_*z*_ PL and SiO_2_ IL. A Keithley 4200-SCS parameter analyzer was used to examine the Al/Tm_*x*_O_*y*_N_*z*_/4H-SiC/Al MOS structure’s capacitance–voltage (*C–V*), conductance-voltage (*G*–*V*), and current–voltage (*I–V*).

## 3 Results and Discussion

Figure 1a shows the GIXRD spectra for the Tm_*x*_O_*y*_N_*z*_ PL subjected to dual-stage (RTA and NA) and single-stage (NA) annealing processes. GIXRD analysis confirmed the formation of the Tm_*x*_O_*y*_N_*z*_ phase, evidenced by diffraction peaks corresponding to the (222), (400), (440), and (622) planes. A notable shift in these peaks toward lower angles was observed for both passivation layers when compared to the reference Tm_2_O_3_ phase (ICDD file no. of 00-041-1090). This angular shift was consistent with lattice expansion, likely induced by the substitution of smaller oxygen ions (1.40 Å^23^) with larger nitrogen ions (1.46 Å^23^) within the crystal structure. Furthermore, compared to the single-stage annealed Tm_*x*_O_*y*_N_*z*_ PL,^24^ the Tm_*x*_O_*y*_N_*z*_ peaks for the dual-stage annealed Tm_*x*_O_*y*_N_*z*_ PL were seen at larger diffraction angles, indicating that more nitrogen ions had been incorporated into the Tm_*x*_O_*y*_N_*z*_ lattice during the single-stage annealing process. Consequently, the single-stage annealed Tm_*x*_O_*y*_N_*z*_ PL exhibited a larger lattice parameter *a* of 10.530 Å, while the value for the dual-stage annealed sample was measured at a slightly lower value of 10.517 Å. It could be inferred from these findings that the dual-stage process enabled a more substantial accumulation of nitrogen at the interface. This nitrogen-rich layer subsequently acted to block oxygen transport from the SiC substrate, which successfully prevented the nucleation of the SiO_2_ IL.

**Figure 1:**
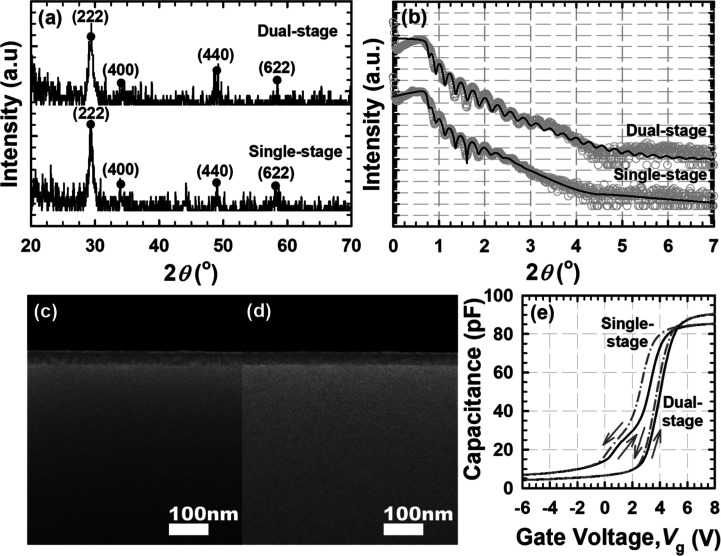
Tm_*x*_O_*y*_N_*z*_ PL (a) GIXRD pattern and (b) XRR curves after single-stage^24^ and dual-stage annealing. Tm_*x*_O_*y*_N_*z*_ PL cross-sectional FESEM images after (c) single-stage^24^ and (d) dual-stage annealing. (e) Bidirectional *C*–*V* curves (1 MHz) for the Tm_*x*_O_*y*_N_*z*_ PL subjected to single-stage^24^ and dual-stage annealing.

This might suggest that in contrast to the single-stage annealed Tm_*x*_O_*y*_N_*z*_ PL, the addition of an extra RTP step in the dual-stage annealing process encouraged a greater diffusion of nitrogen ions to the interface. This is probably because the nitrogen ions caused a quenching effect on the atomic vibration of Si atoms.^25^ The fitted XRR data validated the nitrogen diffusion barrier layer’s suppressive impact on SiO_2_ IL formation (Figure 1b), which showed that the dual-stage annealed Tm_*x*_O_*y*_N_*z*_ PL had a thinner SiO_2_ IL (2.422 nm) compared to the single-stage annealed Tm_*x*_O_*y*_N_*z*_ PL (3.677 nm).^24^ This observation aligned with the aforementioned deduction regarding the disparate distribution of nitrogen ions in the single- and dual-stage annealed Tm_*x*_O_*y*_N_*z*_ PL. The XRR results also revealed that a thinner Tm_*x*_O_*y*_N_*z*_ PL (33.644 nm) was achieved with the dual-stage annealed Tm_*x*_O_*y*_N_*z*_ PL compared to the single-stage annealed Tm_*x*_O_*y*_N_*z*_ PL.^24^ This provided further evidence in favor of the previous finding that the single-stage annealed Tm_*x*_O_*y*_N_*z*_ PL had more nitrogen ions incorporated into the Tm_*x*_O_*y*_N_*z*_, which restricted the densification of the Tm_*x*_O_*y*_N_*z*_ PL. Additionally, cross-sectional FESEM images for the single-stage (Figure 1c) and dual-stage (Figure 1d) annealed Tm_*x*_O_*y*_N_*z*_ PL revealed an average total oxide thickness of 38.686 and 38.159 nm, respectively, measured from 10 different locations. These results exhibited trends consistent with the XRR results.

Evidence from XPS, namely, the N 1s, O 1s, and Tm 4d core level spectra shown in Figure 2a–f verified that nitrogen ions were incorporated into the material’s lattice. In both single- and dual-stage annealed Tm_*x*_O_*y*_N_*z*_ PL, the N 1s core level spectra showed two notable peaks that corresponded to Tm–O–N and Tm–N bonding. It was noticed that the single-stage annealed Tm_*x*_O_*y*_N_*z*_ PL exhibited higher peak intensities for both Tm–O–N and Tm–N bonding compared to the dual-stage annealed Tm_*x*_O_*y*_N_*z*_ PL, suggesting that the single-stage annealed Tm_*x*_O_*y*_N_*z*_ PL has a larger concentration of nitrogen ions.^24^ The N 1s core level spectra revealed that nitrogen integration was more pronounced in the single-stage annealed PL, a conclusion drawn from the lower binding energy measured for its Tm–N and Tm–O–N peaks. In opposition to this trend, the corresponding O 1s spectra for the dual-stage processed sample (Figure 2e) showed Tm–O–N and Tm–O peaks that had shifted to higher binding energies and gained intensity, signaling a greater occupation of *V*_o_ by oxygen ions. As a result, a lower intensity of the *V*_o_ peak at 531.0 eV was observed in the O 1s core level spectra, suggesting that the dual-stage annealed Tm_*x*_O_*y*_N_*z*_ PL had a lower concentration of *V*_o_. Two mechanisms could be proposed to explain these findings. First, the dual-stage process appeared to facilitate nitrogen ion segregation at the interface, which created a thicker barrier that impeded oxygen transport toward the 4H-SiC substrate. This forced oxygen ions to diffuse outward, annihilating *V*_o_ in the Tm_*x*_O_*y*_N_*z*_. The postulation of nitrogen ions segregating at the Tm_*x*_O_*y*_N_*z*_/4H-SiC interface was in agreement with previous reported work related to low-pressure chemical vapor deposited SiO_2_ on 4H-SiC substrate that was subjected to post-oxidation annealing using 10% of nitric oxide gas at 1300 °C for 30 min, whereby XPS and hard XPS measurements have revealed that nitrogen ions existed at the SiO_2_/4H-SiC interface.^26^ A second hypothesis suggested the single-stage PL’s prominent *V*_o_ peak originated from a higher level of nitrogen incorporation into the crystal lattice, which could have stabilized such defects.^24^ This incorporation has caused the release of oxygen ions from the Tm_*x*_O_*y*_N_*z*_ lattice to achieve charge neutrality. Besides, the Tm–O–H peak at 532.1 eV was detected in both single- and dual-stage annealed Tm_*x*_O_*y*_N_*z*_ PL due to the employment of FG. It was perceived that the single-stage annealed Tm_*x*_O_*y*_N_*z*_ PL exhibited a higher intensity of the Tm–O–H peak, likely because of the absence of the RTA step, whereby before NA started, no nitrogen ions had diffused to the interface. This made it possible for hydrogen ions to readily permeate to the interface during the single-stage annealing process, which led to a more intense Tm–O–H spectral peak.^24^ Figure 2c,f presents the Tm 4d spectra for the two passivation layers, revealing a systematic shift toward higher binding energies for the Tm–O, Tm–O–N, and Tm–N chemical states in the dual-stage annealed sample. This trend aligned with the spectral changes observed in the corresponding O 1s and N 1s core levels.

**Figure 2:**
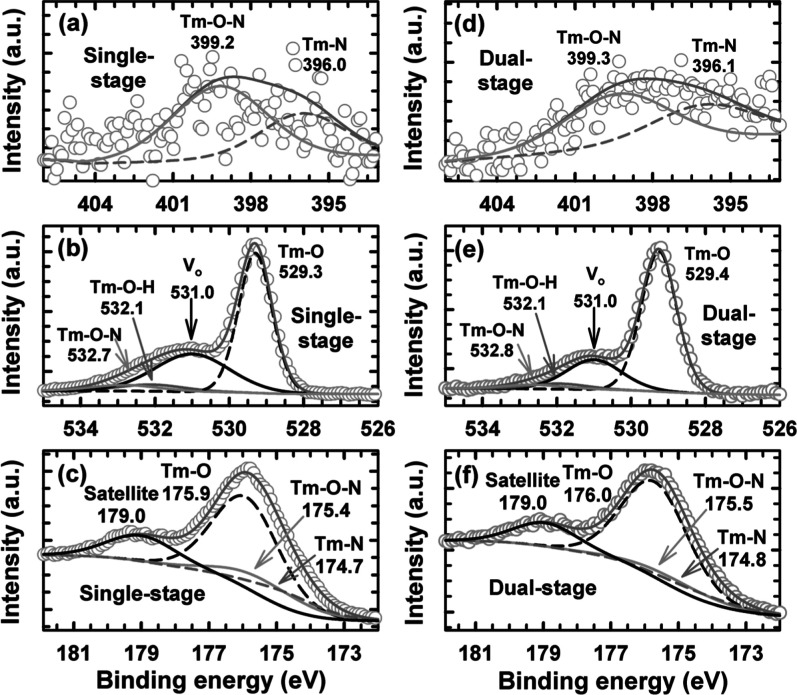
Comparison of the N 1s, O 1s, and Tm 4d XPS core level spectra for the Tm_*x*_O_*y*_N_*z*_ PL after single-stage (a–c)^24^ and dual-stage (d–f) annealing.

The *C*–*V* characteristics of the investigated Tm_*x*_O_*y*_N_*z*_ PL, shown in Figure 1e, displayed an irregular *C–V* curve for the single-stage annealed Tm_*x*_O_*y*_N_*z*_ PL within the applied gate voltage range of 0 to +2 V. This suggested that the single-stage annealed Tm_*x*_O_*y*_N_*z*_ PL contained a high concentration of defects, likely related to the *V*_o_, as confirmed by the O 1s core level spectrum (Figure 2b).^24^ The *C*–*V* curves also indicated that the single-stage annealed Tm_*x*_O_*y*_N_*z*_ PL exhibited a smaller positive flat band voltage (*V*_FB_) than the dual-stage annealed Tm_*x*_O_*y*_N_*z*_ PL, suggesting that more negatively charged traps were formed in the dual-stage annealed Tm_*x*_O_*y*_N_*z*_ PL. This observation was consistent with the calculated effective oxide charge (*Q*_eff_), which showed that the dual-stage annealed Tm_*x*_O_*y*_N_*z*_ PL had a higher negatively charged *Q*_eff_ of 5.323 × 10^12^ cm^–2^ compared to the single-stage annealed Tm_*x*_O_*y*_N_*z*_ PL (4.094 × 10^12^ cm^–2^).^24^ These results further confirmed that the single-stage annealed Tm_*x*_O_*y*_N_*z*_ PL was composed of more positively charged *V*_o_, contributing to a reduction in the overall number of negative traps in this PL.^24^ In contrast, the higher negative *Q*_eff_ value obtained for the dual-stage annealing process reinforced the idea that the additional RTA step triggered the more negatively charged nitrogen ions building up at the interface, which prevented a SiO_2_ IL from forming. The inhibition of SiO_2_ IL formation using the dual-stage annealing process was further confirmed by the *C*–*V* measurements, where the dual-stage annealed Tm_*x*_O_*y*_N_*z*_ PL attained a higher *k* value of 13.1 compared to the single-stage annealed Tm_*x*_O_*y*_N_*z*_ PL (*k* = 12.2).^24^

Besides, the bidirectional *C*–*V* curves revealed that the dual-stage annealed Tm_*x*_O_*y*_N_*z*_ PL exhibited a narrower hysteresis than the single-stage annealed Tm_*x*_O_*y*_N_*z*_ PL (Figure 1e), indicating that the former sample had a lower slow trap density (STD; 4.24 × 10^11^ cm^–2^) than the latter sample (10.87 × 10^11^ cm^–2^).^24^ This observation suggested that during forward bias, the capturing of injected electrons was limited by the presence of more negatively charged nitrogen ions at the interface between the dual-stage annealed Tm_*x*_O_*y*_N_*z*_ PL and the 4H-SiC substrate, resulting in a lower density of electrons being released when reverse bias was applied. For the single-stage annealed Tm_*x*_O_*y*_N_*z*_ PL, the formation of a thicker SiO_2_ IL contributed to the formation of more positively charged *V*_o_ near to the interface, where these *V*_o_ acted as slow traps contributing to trapping and detrapping during forward and reverse bias, respectively. Furthermore, Terman’s high–low frequency, and conductance methods were used to examine the interface quality of these samples. The interface trap density (*D*_it_) values that were determined using these techniques are shown in Figure 3a–c. Additionally, Figure 3d showed the typical corrected *C*–*V* and *G*–*V* curves, recorded at various frequencies for the dual-stage annealing Tm_*x*_O_*y*_N_*z*_ PL, which were used to calculate *D*_it_ using high–low frequency and conductance methods, respectively. A consistent trend was observed between the high–low frequency and conductance methods, suggesting that the dual-stage annealed Tm_*x*_O_*y*_N_*z*_ PL exhibited better interface quality, as indicated by its lower *D*_it_ compared to the single-stage annealed Tm_*x*_O_*y*_N_*z*_ PL.^24^ Nonetheless, the *D*_it_ extracted using Terman’s method showed a different trend, likely due to the high density of slow charge trapping and detrapping in the single-stage annealed Tm_*x*_O_*y*_N_*z*_ PL, which led to an overestimation of band bending.^27^ The higher calculated STD value in the single-stage annealed Tm_*x*_O_*y*_N_*z*_ PL compared to the dual-stage annealed Tm_*x*_O_*y*_N_*z*_ PL further supported the presence of slow charge trapping and detrapping.^24^ The current density-electric field (*J*–*E*) characteristics of these PL were presented in Figure 3e, revealing that the additional RTA step helped improve the *J* of the dual-stage annealed Tm_*x*_O_*y*_N_*z*_ PL. Nevertheless, the slightly higher *E*_B_ demonstrated by the single-stage annealed Tm_*x*_O_*y*_N_*z*_ PL could be attributed to the formation of a thicker SiO_2_ IL. Despite this, the detrimental effect of the low-*k* SiO_2_ IL formation on a reduction in the overall *k* value was observed for the single-stage annealed Tm_*x*_O_*y*_N_*z*_ PL.^24^ Therefore, the demonstration of an enhanced dual-stage annealed Tm_*x*_O_*y*_N_*z*_ PL with improved *k*, reduced *D*_*it*_, and lower *J* represented a valuable step toward meeting the multifaceted performance requirements for next-generation 4H-SiC MOS-based devices.^15^

**Figure 3:**
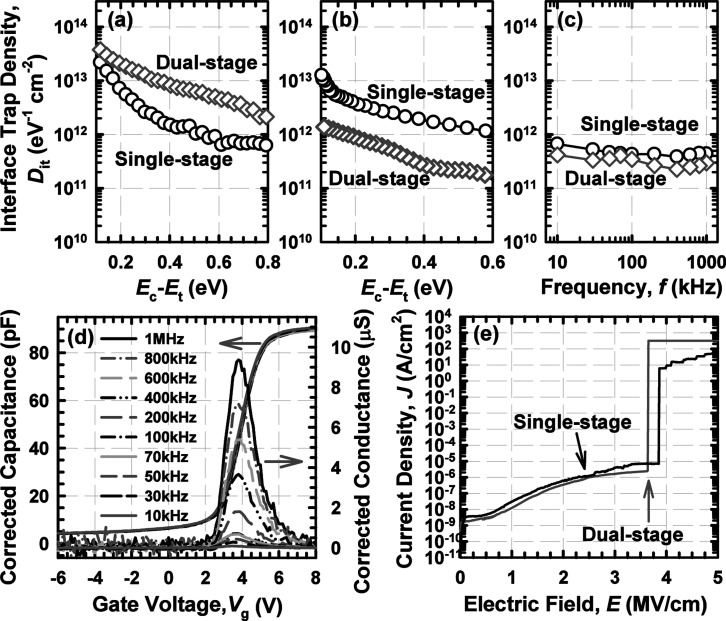
(a) Terman’s technique, (b) high–low frequency capacitance method, and (c) conductance method used to compute the *D*_it_ of the Tm_*x*_O_*y*_N_*z*_ PL after single-stage^24^ and dual-stage annealing. Corrected *C*–*V* and *G*–*V* curves (d). *J*–*E* characteristic of the Tm_*x*_O_*y*_N_*z*_ PL subjected to single-stage^24^ and dual-stage annealing (e).

## 4 Conclusion

This study enhanced the performance of Tm_*x*_O_*y*_N_*z*_ as a PL for MOS devices based on 4H-SiC using a novel dual-stage annealing process involving RTA in ambient N_2_ followed by NA in ambient FG-O_2_-FG ambient. The additional RTA step improved MOS characteristics, resulting in a higher *k* value (13.1), lower STD (4.24 × 10^11^ cm^–2^), and lower *D*_it_. GIXRD and XPS measurements revealed that these improvements were due to nitrogen ion buildup at the Tm_*x*_O_*y*_N_*z*_/4H-SiC interface and the reduction of *V*_o_, leading to a thinner SiO_2_ IL thickness (2.422 nm). Additionally, an improvement in *J* was perceived for the dual-stage annealing of the Tm_*x*_O_*y*_N_*z*_ PL.
